# Retinopathy of Prematurity among Preterm Newborn Admitted to the Neonatal Care Unit in a Tertiary Care Centre: A Descriptive Cross-sectional Study

**DOI:** 10.31729/jnma.8117

**Published:** 2023-04-30

**Authors:** Pradeep Bastola, Swapnil Madhukar Parchand, Anil Babanrao Gangwe, Sheeksha Bastola, Deepshikha Agrawal

**Affiliations:** 1 Department of Ophthalmology, Chitwan Medical College, Bharatpur, Nepal; 2 MGM Eye Institute, Raipur, India; 3 Gandaki Medical College, Nayabazaar Rd, Pokhara, Nepal

**Keywords:** *blood transfusion*, *low birth weight*, *oxygen*, *preterm births*, *retinopathy of prematurity*

## Abstract

**Introduction:**

World Health Organization has identified retinopathy of prematurity as an important cause of preventable childhood blindness. The presentation of retinopathy of prematurity is varied and differs in the developed and developing worlds. The study aimed to find out the prevalence of retinopathy of prematurity among preterm newborn admitted to the Neonatal Care Unit in a tertiary care centre.

**Methods:**

A descriptive cross-sectional study was conducted among preterm newborn admitted to the Neonatal Care Unit after receiving ethical approval from the Institutional Review Committee (Reference number: IEC/MGMEI/I/2021/66). The study was conducted from 15 December 2021 to 17 February 2022. Basic demographic data, risk factors, clinical characteristics, and prevalence of retinopathy of prematurity were noted. Convenience sampling was done. Point estimate and 95% Confidence Interval were calculated.

**Results:**

Among 204 participants, retinopathy of prematurity was found in 118 (57.84%) (51.06-64.62, 95% Confidence Interval) in at least one eye. Early treatment retinopathy of prematurity type 2 in 82 (69.49%) was the commonest one severity-wise. Supplemental oxygen was given to 118 (100%) cases, and low birth weight was present in 109 (92.37%) cases.

**Conclusions:**

The prevalence of retinopathy of prematurity was found to be higher in other similar studies done in similar settings. The screening and treatment for the retinopathy of prematurity require a dedicated trained team of ophthalmologists, vitreo-retina specialists, paediatricians, and neonatologists with well-developed facilities for retinopathy of prematurity clinics.

## INTRODUCTION

The World Health Organization (WHO) has identified retinopathy of prematurity (ROP) as an important cause of preventable and treatable blindness worldwide.^1^ The presentation of ROP varies according to the geography with the prevalence, and severity of ROP being more in the low resource settings.^1,2^

ROP affects immature vasculature of premature infants' eyes. This disease can appear in mild and aggressive forms. Visual complications rarely result in the milder form of ROP, while the aggressive type is along with neovascularization and develops into retinal detachment and blindness.^3,4^ The prevalence of ROP is increasing due to the survival of smaller and younger infants.^5,6^

This study aimed to find out the prevalence of retinopathy of prematurity among preterm newborn admitted to the Neonatal Care Unit in a tertiary care centre.

## METHODS

This descriptive cross-sectional study was conducted at MGM Eye Institute, Raipur, India from 15 December 2021 to 17 February 2021 for a period of over 2 months. Ethical approval was obtained from the Institutional Review Committee (Reference number: I EC/MGMEI/I/2021/66). Preterm were included in the study. The informants not providing informed consent, and critically ill preterm not suitable for examination were excluded from the study. A convenience sampling technique was used. The sample size was calculated using the formula:


$$\begin{array}{ll} \text{n} & ={\text{Z}}^{2}\times \frac{\text{p}\times \text{q}}{{\text{e}}^{\text{2}}}\\ & =1.96^{2}\times \frac{0.50\times 0.50}{0.07^{2}}\\ & =197 \end{array}$$

Where,

n = minimum required sample size
Z = 1.96 at 95% Confidence interval (CI)
p = prevalence taken as 50% for maximum sample size calculation
q = 1-p
e = margin of error, 7%

The calculated sample size was 197. However, 204 participants were included in the study.

All newborn were screened as per the latest National Neonatal Forum guidelines, India (NNF clinical practice guidelines-retinopathy of prematurity). The level of prematurity of screened newborn in the present study was defined as extreme preterm (<28 weeks, very preterm (28-32 weeks), moderately preterm (33-34 weeks), late preterm (35-37 weeks), and term (>37 weeks).^7^

Baseline characteristics noted for all newborn were the mother's name, sex, gestational age, birth weight, age at the time of first screening, and being small/appropriate for gestational age (SGA/AGA). Various risk factors were analyzed in the study, which included respiratory distress syndrome (RDS), neonatal hyperbilirubinemia (NNH), neonatal sepsis (NNS), birth asphyxia, necrotizing enterocolitis (NEC), multiple gestation, shock, Rh incompatibility, hypocalcemia, hypothermia, patent ductus arteriosus (PDA), apnoea, hypoglycemia, and gastrointestinal haemorrhage (GIH), blood transfusion and broad-spectrum antibiotics use for any infection.

The findings from the ROP screening were noted in a standard format used by MGM Eye Institute, and ROP was staged and classified as no ROP, mild ROP was defined as not requiring treatment, and severe ROP cases were defined as requiring laser treatment/anti-vascular endothelial growth factor (VEGF) or surgery. Severe ROP included all newborn of threshold ROP as defined by CRYO-ROP, threshold type I ROP as defined by early treatment retinopathy of prematurity (ETROP)^4,8,9^ study, aggressive posterior ROP (APROP), and all cases diagnosed as advanced ROP (Stage IV and V) at the time of screening. In the current study international classification for ROP (ICROP) was also used to stage and classify ROP.^4,8,9^ A Retinal camera (RetCam) shuttle connected to a laptop was used to take the clinical photograph of a baby needing intervention or follow-up comparisons.

Data were entered and analyzed using IBM SPSS Statistics version 26.0. Point estimate and 95% CI were calculated.

## RESULTS

Among 204 participants, 118 (57.84%) (51.06-64.62, 95% CI) study subjects had retinopathy of prematurity changes in at least one eye. Early treatment retinopathy of prematurity (ETROP) type 2 was the commonest ROP, seen in 82 (69.50%) of the study subjects followed by severe ROP or ETROP 1 in 36 (30.50%). Similarly, stage 1 ROP was observed in 63 (53.38%) patients. Retinal involvement-wise, Zone 2 with 69 (58.47%) was most commonly involved (Table 1).

**Table 1:** Types, severity, staging, and zone-wise involvement (n = 118).

Variables	n (%)
ETROP Type 1 (Severe ROP)	8 (6.78)
ETROP Type 2 (Mild ROP)*	82 (69.49)
APROP	19 (16.10)
Hybrid Disease (Severe ROP)	9 (7.63)
**Stage of ROP**	
Immature Retina	19 (16.10)
Stage 1	63 (53.39)
Stage 2	24 (20.34)
Stage 3	10 (8.47)
Stage 4A	2 (1.69)
**Retinopathy of prematurity zone wise**	
Zone 1	34 (28.81)
Zone 2	69 (58.47)
Zone 3	15 (12.71)

Supplemental oxygen 118 (100%), low birth weight 109 (92.37%), twin pregnancy 50 (42.37%) blood transfusion 38 (32.20%), sepsis 30 (25.42%), and birth asphyxia 20 (16.94%) were the major risk factors to develop retinopathy of prematurity in the study subjects, however, none of the study subjects had hypocalcemia, patent ductus arteriosus, Rh incompatibility, and gastrointestinal haemorrhage. Extremely low birth weight (birth weight less than 1500 g in 84 (71.18%) was a very important observation in patients with ROP. The clinical characteristics of the study subjects with retinopathy of prematurity (ROP) in the present study are summarized below (Table 2).

**Table 2:** Clinical characteristics (n = 118).

Variables	n (%)
Supplemental oxygen requirement	118 (100)
Low birth weight	109 (92.37)
Blood transfusion requirement	38 (32.20)
Infection or sepsis	30 (25.42)
Birth asphyxia	20 (16.95)
Ventilator support	12 (10.17)
Shock	12 (10.17)
Hypothermia	10 (8.47)
**Twin pregnancy**	
Twin preterm newborn	50 (42.37)
Single preterm baby	68 (57.63)
Rh incompatibility	-
No history of hypocalcemia	100 (100)
No history of gastrointestinal haemorrhage	118 (100)
**Respiratory distress syndrome (RDS) in study participants**	
Study subjects without RDS	116 (98.31)
Study subjects developing RDS	2 (1.69)
**Study participants needing intervention**	
Treated ROP subjects	36 (30.51)

Gender wise males 68 (57.62%) developed ROP more than females. The mean gestational age of the study participants at birth was 30.45±2.44 weeks. The mean birthweight of the study participants was 1340.66±355.10 gm. The mean gestational age of the study participants during the first ROP screening was 33.49±2.43 weeks. Similarly, the mean age of the study participants when ROP got diagnosed was 34.88±2.48 weeks. The mean haemoglobin levels of the preterm newborn in the study were 10.91±2.22 g/dl. Extreme preterm and very preterm newborn were 36 (30.50%), and 51 (43.22%) respectively (Table 3).

**Table 3:** Demographic profile (n = 18)

Variables	n (%)
**Gender distribution**	
Male	68 (57.62)
Female	50 (42.38)
**Gestational age of participants at birth in weeks**	
Extreme preterm newborn	36 (30.50)
Very preterm newborn	51 (43.22)
Moderately preterm newborn	14 (11.87)
Late preterm newborn	17 (14.41)
Term newborn	-

## DISCUSSION

The prevalence of ROP in the current study was 57.8%. The prevalence of mild ROP was 82 (69.50%). According to the international classification for retinopathy of prematurity (ICROP) stage 1, ROP was observed in 53.4% of the study participants. Retinal involvement-wise, zone 2 with 69 (58.5%) was the most commonly involved zone of the retina in the current study in the subjects with ROP. In two of the studies from India, the prevalence of ROP was 30% and 24,1%.^6,10^ These study findings from India were not comparable with our study, as in our study the overall prevalence of any ROP was much higher.^6,10^ However, in a study done in KSA^11^ the incidence of ROP was 56%, which was comparable with the findings of our study.

In the current study, a mild form of ROP was seen in 69.50% of the total study participants which correlated well clinically with the other study findings like the stage of ROP and zone of the retina. In the present study, 30.50% study participants had severe ROP, which warranted intervention. In the severe ROP group, classic ROP was diagnosed in 14.40% study subjects, whereas 16.10% of the study participants were diagnosed with aggressive posterior ROP (APROP). These findings of the study are well supported by the associated risk factors and clinical characteristics in the study participants. In a study from India^6^ the prevalence of severe ROP was found to be 14.20% of which the classic form of ROP constituted 55.50% of all severe ROP cases, and APROP 27.70%, which did not correlate with our study. Similarly, a study from Turkey reported the prevalence of any stage of ROP to be 27%, and the prevalence of severe ROP to be just 6.70%^12^ which again did not compare well with our study findings. The high prevalence of any stage ROP and severe ROP in the current study could be due to the smaller sample size, persistent Corona virus disease 2019 pandemic (third wave) compromising neonatal care, and relatively smaller, sicker, and younger participants in the study.

In the present study, supplemental oxygen 100%, low birth weight (92.4%), twin pregnancy (42.37%), blood transfusion (32.20%), sepsis (25.42%), and birth asphyxia (16.94%) were the major risk factors to develop retinopathy of prematurity in the study subjects. In addition, shock and ventilator use among the study participants were also significant factors accounting for (10.10%) of the study participants. These risk factors have been well studied in the past and have a strong association with the development of any stage of ROP. This finding of our study was comparable with various studies done in the past across the globe.^6,10-13^

In the current study, there was a positive correlation between oxygen supplementation either with a nasal cannula or mechanical ventilation, and the development of any stage of ROP. These findings from our study were comparable with other studies which confirmed the association between the risk of ROP and the use of mechanical ventilation and continuous positive airway pressure (CPAP).^13-15^ Likewise mean age of the study participants when ROP got diagnosed in the current study was 34.88±2.48 weeks, and the mean haemoglobin levels of the study subjects were 10.91±2.22-g/dl. Low levels of haemoglobin and the mean gestational age at which ROP was diagnosed correlated and compared well with the studies from the past.^13-15^

In our study, none of the study participants had a history of intraventricular haemorrhage, thrombocytopenia, Rh incompatibility, gastrointestinal haemorrhage, hypocalcemia, or patent ductus arteriosus. These above-mentioned risk factors have been associated with the development of ROP; this finding of our study was not comparable with studies done elsewhere.^13^-^16^"^18^

In the current study, males (57.62%) were more predominant than females, this finding of the study compared well with a study done elsewhere.^13^ Mean gestational age of the study participants at birth in the current study was 30.45±2.44 weeks. Similarly, the mean birthweight of the study participants was 1340.66±355.10 g. Extremely low birth weight (birth weight less than 1500 g) was an important risk factor in 84 (71.18%) study participants to develop any ROP in the current study, in addition, 28 (23.72%) of the study participants with severe ROP were less than 1500 grams in weight. Low birth weight and extremely low birth weight has been identified as a very important risk factor to develop ROP in the literature. These findings of the study correlated and compared very well with the existing literature on ROP.^1,2,12"18^

The higher prevalence of any ROP, and severe ROP in the current study could be largely attributed to the extremely low birth weight of the study participants, young gestational age, lesser mean haemoglobin levels, supplemental oxygen therapy, blood transfusion, sepsis, asphyxia, shock, and ventilator use.

This study was carried out during the COVID-19 pandemic, which must have had an impact on the health"seeking behavior of the informants as well as difficulty from healthcare providers. The study also had a smaller sample size, a prospective study with a larger sample size will help to explore more regarding the incidence, and risk factors related to ROP in preterm newborn.

## CONCLUSIONS

The prevalence of ROP was higher than the other studies done in similar settings. Mild ROP still is the commonest form of ROP in most preterm newborn. The screening and treatment for the ROP require a dedicated trained team of Ophthalmologists, vitreo" retina specialists, paediatricians, and neonatologists with well"developed facilities for ROP treatment.
